# A Fermented Food Product Containing Lactic Acid Bacteria Protects ZDF Rats from the Development of Type 2 Diabetes

**DOI:** 10.3390/nu11102530

**Published:** 2019-10-20

**Authors:** Miriam Cabello-Olmo, Maria Oneca, Paloma Torre, Neira Sainz, María J. Moreno-Aliaga, Elizabeth Guruceaga, Jesús Vicente Díaz, Ignacio J. Encio, Miguel Barajas, Miriam Araña

**Affiliations:** 1 Biochemistry Area, Department of Health Science, Public University of Navarre, 31008 Pamplona, Spain; 2 Nutrition and Bromatology area, Department of Natural Sciences, Public University of Navarre, 31006 Pamplona, Spain; 3 Department of Nutrition Food Sciences and Physiology, Center for Nutrition Research, University of Navarra, 31008 Pamplona, Spain; 4 CIBERobn Physiopathology of Obesity and Nutrition, Centre of Biomedical Research Network, ISCIII, 28029 Madrid, Spain; 5 IDISNA, Navarra’s Health Research Institute, 31008 Pamplona, Spain; 6 Bioinformatics Platform, Center for Applied Medical Research, University of Navarra, 31008 Pamplona, Spain; 7 Pentabiol S.L., Polígono Noain-Esquiroz s/n, 31191 Pamplona, Spain

**Keywords:** diabetes, fermented food, gut microbiota, lactic acid bacteria, postbiotic, probiotic

## Abstract

Type 2 diabetes (T2D) is a complex metabolic disease, which involves a maintained hyperglycemia due to the development of an insulin resistance process. Among multiple risk factors, host intestinal microbiota has received increasing attention in T2D etiology and progression. In the present study, we have explored the effect of long-term supplementation with a non-dairy fermented food product (FFP) in Zucker Diabetic and Fatty (ZDF) rats T2D model. The supplementation with FFP induced an improvement in glucose homeostasis according to the results obtained from fasting blood glucose levels, glucose tolerance test, and pancreatic function. Importantly, a significantly reduced intestinal glucose absorption was found in the FFP-treated rats. Supplemented animals also showed a greater survival suggesting a better health status as a result of the FFP intake. Some dissimilarities have been observed in the gut microbiota population between control and FFP-treated rats, and interestingly a tendency for better cardiometabolic markers values was appreciated in this group. However, no significant differences were observed in body weight, body composition, or food intake between groups. These findings suggest that FFP induced gut microbiota modifications in ZDF rats that improved glucose metabolism and protected from T2D development.

## 1. Introduction

Type 2 diabetes (T2D) is characterized by a chronic hyperglycemia preceded by a deranged insulin sensitivity and/or synthesis. This chronic non-communicable disease accounts for the vast majority of cases of diabetes, more than 90% [1], being one of the most prevalent illnesses. Importantly, T2D is associated with over 70% of global deaths [2]. Genetic factors contribution is unlikely to be responsible for the increasing T2D incidence because of the genome stability, although physicians and the scientific community have also focused on environmental factors [3,4]. One possible explanation hypothesizes the role of microorganisms that coexist with us [5]. The gut microbiota (GM) is the bigger reservoir (10^14^ microbes) [5] and is considered an organ because of its crucial metabolic and defense competences [6]. Its anomalous distribution or activity, named dysbiosis [7], has been related to a wide group of illnesses and physiopathological conditions [8]. Specifically, there is evidence that diabetes occurs in association with a compromised gut environment and a large body of research highlights a plausible connection between T2D and GM [9,10,11]. For instance, a compromised abundance of beneficial bacteria [12] or increase of infrequent species [13] have been reported in T2D and obese individuals.

The GM displays some plasticity and different strategies resulted in marked changes. Among the studied approaches, dietary modulation was successful in causing significant changes in the GM [13,14]. Probiotics, prebiotics, symbiotics, and fermented foods are known to confer health benefits on the host by improving GM performance and have been proposed as novel clinical strategies for T2D [6,15,16]. For instance, probiotic supplementation with *Lactobacillus* G15 and Q14 showed improved glucose tolerance in streptozotocin (STZ)-induced type 2 diabetic rats [17]. Human intervention studies have also shown good results. A crossover trial with prediabetic individuals reported a reduced insulin resistance after kimchi consumption [18], and a randomized controlled trial in T2D subjects supplemented with a fermented milk with the probiotic *Lactobacillus casei* strain Shirota showed a healthier gut ecosystem with strengthened gut barrier function along with modulation of microbial communities [19].

The most widespread fermented products are dairy products like yogurt, kefir, or dahi. Nonetheless, other food matrixes like fruits, vegetables, or cereals have also been studied and showed many beneficial effects on health [20]. Indeed, some attractive advantages over dairy products have been described for them [21]. In the present study we have tested the effectiveness of a non-dairy fermented food product (FFP) in preventing the T2D and obese phenotype developed by the Zucker diabetic fatty (ZDF) rat model. This murine model presents a mutation in the leptin receptor accompanied with an enhanced β-cell destruction and impaired glucose homeostasis, and is a widely used model for T2D studies [22,23]. Previous research reported the effectiveness of probiotics in the attenuation of the diabetic and obese phenotype in ZDF rat model [24] and other rodent models [25,26]. However, few researchers have tested the efficiency of fermented food on the diabetic phenotype and previous works have only focused on dairy products [27]. On top of this, the literature on probiotic microorganisms indicates that the functional attributes of the cells are to a large degree dependent on the strain [28,29]. Therefore, an individual characterization should be performed for each study product. Our fermented food product derives from a fermented feed, which has previously demonstrated the ability to improve health and wellness in farm animals (HEALTHSTOCK Ref. 733627; https://cordis.europa.eu/project/rcn/206082/factsheet/es). Consequently, FFP has been used in a controlled in vivo study (ZDF rat model) in order to demonstrate its benefit in glycemic control and in comorbidities derived from hyperglycemia.

## 2. Materials and Methods 

### 2.1. Product Description

The fermented food product tested is a plant-based food product primarily composed by soya flour, alfalfa meal, and barley sprouts along with other minor components (including skimmed milk powder). The FFP is defined as a non-probiotic product classified as fermented food [29]. During the production process a combination of specific LABs and non-bitter beer yeast is incorporated to the raw materials and a classical fermentation process is performed. The FFP has been produced using standard culture medium Tryptic Soya Agar (TSA) in microaerophilic conditions at 37 °C until microorganisms’ concentration achieve at least 10^9^ microorganisms/mL in an exponential growth phase or close to the stationary phase. The manufacturing as well as the pool of microorganisms intentionally added are responsible for the viable microorganisms and composition of the final product (Tables S1 and S2). The FFP is presented as a dry granulated product, with an average particle size ranging from 4 to 12 mm (Figure S1) with a moisture content of 12.8% and a pH of 4.4. The metagenomics analysis revealed that Firmicutes is the most predominant phylum (38.7%), followed by Proteobacteria (26.7%), Bacteroidetes (18.3%), Actinobacteria (14.5%), and lately TM7 (1.8%). At genus level, *Lactobacillus* are the most predominant accounting for more than 6% of identified species. 

### 2.2. Animals and Experimental Design

Eleven weeks-old male ZDF rats (*n* = 16) (Charles River Laboratories) were acclimated for five weeks and housed in a controlled environment (a room with constant temperature and humidity under a 12:12 h light-dark cycle) with ad libitum access to food (standard rodent chow) and water. Animals were randomly divided and allocated into two groups: A control group (C group) (*n* = 8) and a group supplemented with FFP (T group) (*n* = 8). ZDF rats were housed at four animals per cage. After the acclimatization period, all animals were given hypercaloric diet (HD) (TD.06416; Envigo) until the end of the study, which lasted 31 weeks (see composition of HD in Table S2). The T group was additionally fed with FFP (200 g per cage and week). See the experimental design scheme (Figure S2). The glucose uptake assay, the insulin positive cell quantification such as the analysis of the microbiota were analyzed by blinded investigators.

Animal procedures were performed in accordance with the “Principles of Laboratory Animal Care” formulated by the National Society for Medical Research and the “Guide for the Care and Use of Laboratory Animals” prepared by the Institute of Laboratory Animal Resources, Commission on Life Science, National Research Council, and published by the National Academy Press, revised 1996. All animal procedures were approved by the Institutional Committee on Care and Use of Laboratory Animals (CEEA, University of Navarra) (Protocol number: CEEA/117-15).

### 2.3. Fasting Blood Glucose and Intraperitoneal Glucose Tolerance Test 

Animals were fasted 24 h and blood samples were collected from the tip of the tail vein in order to determine blood glucose levels by using a glucometer (Accu-chek Aviva, Roche, Basel, Switzerland). Fasting blood glucose (FBG) was recorded once a week. 

For glucose tolerance test (GTT) determination, 24 h fasted animals received glucose (Baxter, Valencia) intraperitoneally (1.5 g/kg of body weight) and glycemia was determined as described for FBG at different time points (baseline, 20, 40, 60, 90, 120, and 150 min) after glucose injection. GTT was conducted at one and two months after the start of the study. The area under the curve (AUC) of glucose values was assessed for each group from 0 to 150 min post glucose injection. 

### 2.4. Body Weight, Food Intake and Body Composition 

Body weight (BW) was measured once a week with an electronic balance. HD food intake was monitored weekly for 12 weeks. The weekly HD intake mean was estimated for the two experimental groups as grams per week and animal. Also, body composition was determined for each group (*n* = 4 in C group; *n* = 5 in T group) using nuclear magnetic resonance (EchoMRI, EchoMedical Systems, Houston, TX, USA) at the end of the study.

### 2.5. Analysis of Functional Properties: C-peptide Synthesis and Intestinal Glucose Uptake Assays

Blood C-peptide concentration (ng mL^−1^) was quantified with a commercial C-Peptide ELISA kit (Crystal Chem Europe) at time points four and seven months from the beginning of the study. HOMA-IR and HOMA-β were determined at the end of the study. HOMA-IR was calculated by the formula: HOMA-IR = serum C-peptide (ng mL^−1^)*blood glucose (mmol L^−1^)/22.5; and HOMA-β was calculated by the formula: HOMA-β = 20*serum C-peptide (ng mL^−1^)/(blood glucose (mmol L^−1^) − 0.35).

The effects of in vivo FFP supplementation on the uptake of α-Methyl-D-glucoside (α-MG), a SGLT-1 specific substrate, were determined on everted jejunal rings obtained from the animals as previously described [30]. Briefly, at the end of the study, animals were sacrificed, the jejunum obtained, and groups of six rings were incubated at 37 °C for 15 min under continuous shaking in Krebs-Ringer-Tris (KRT) solution gassed with O_2_. The solution contained 1 mM α-MG and 0.0025 μCi mL^−1^ of [^14^C] α-MG (Ge Healthcare, Little Chalfont, UK). At the end of the incubation period, rings were removed from the medium, weighed, and the accumulated substrate was extracted from the rings for 15 h in 0.1 M HNO_3_ at 4 °C. Finally, duplicate aliquot samples were taken for liquid scintillation counting. α-MG uptake was estimated from the relationship between the counts per minute recorded for the incubation medium and the counts per minute obtained for the HNO_3_ aliquots and expressed as micromoles of α-MG per gram of wet weight (w.w.) per 15 min.

### 2.6. Lipid Profile and Hepatotoxicity Markers

In order to determine the lipid profile, fasting blood samples were extracted from the dorsal pedal vein under anesthesia (5% isoflurane in oxygen) at baseline, two, four, and seven months of the study. Samples were centrifuged 15 min at 13,000 rpm and stored at −80 °C for biochemical analysis. Also, serum total cholesterol (TC), high-density lipoprotein (HDL-C), low-density lipoprotein (LDL-C), triglycerides (TG) and aspartate and alanine amino-transferases (AST and ALT, respectively) were analyzed (Cobas c311 analyzer, Roche, Basel, Switzerland). The atherogenic index (AI) was estimated using the formula log(TG/HDL-C) as previously described [31].

### 2.7. Tissue Collection and Histological Analysis

After 31 weeks of study, o/n fasting animals were sacrificed by decapitation. The pancreas and fat tissues (retroperitoneal, epidydimal, mesenteric, subcutaneous, and brown fat) were immediately removed, weighted, and fixed in 10% buffered formalin.

Fixed pancreas samples were embedded in paraffin blocks, cut at a thickness of 3 µm and analyzed by immunohistochemistry (*n* = 4 for each group). Immunolabelling was performed with an antibody against insulin (dilution 1:8000, A0564 Dako) and a secondary antibody labeled with HRP (dilution 1:100, P0141 Dako). All sections were observed under an optical microscope using the 10× objective lens (Olympus CH, Shinjuku, Japan) and insulin positive cells were counted. Nine serial sections were analyzed for each pancreas. 

The total area (mm^2^) of the analyzed sections was calculated. For this purpose, slides containing the stained histological samples were digitized (APERIO CS2, Leica Biosystems, San Diego, CA, USA) and images were analyzed using the ImageJ 1.52 software. The results of the quantification are shown as insulin-positive cells per pancreas area (insulin positive cells/mm^2^) at the end of the study.

### 2.8. Faecal Microbiota Analysis

Rat feces were collected at six months of study and immediately frozen at −80 °C for the purpose of metagenomic analysis. 16S rRNA sequences obtained were filtered following quality criteria of the OTU processing pipeline LotuS (release 1.58) [32]. This pipeline includes UPARSE de novo sequence clustering [33], removal of chimeric sequences and phix contaminants for the identification of operational taxonomic units (OTUs), and OTU abundance matrix generation. Finally, taxonomy was assigned using BLAST [34] and HITdb [35] achieving up to species-level sensitivity. The abundance matrices were first filtered and then normalized in R/Bioconductor [36] at each classification level: OTU, species, genus, family, order, class, and phylum. Briefly, taxa were discarded for future analysis when less than four reads were obtained in more than 50% of the samples of both experimental conditions, and a global normalization was performed using the library size as a correcting factor and log2 data transformation. Linear models for microarray data (LIMMA) [37] was used to identify taxa with significant differential abundance between experimental conditions. The selection criteria was based on an FDR cut-off (FDR < 0.05). Further clustering analyses and graphical representations were performed using R/Bioconductor [36].

### 2.9. Statistical Analysis

Statistical analysis was performed with the SPSS 22.0 for windows software package. Normality and variances homogeneity were tested with the Shapiro–Wilk and Levene tests, respectively. For values showing normal distribution, comparisons were carried out using unpaired and paired Student’s T-test and in case of non-normal distribution, U-Mann-Whitney test. Results are expressed as mean ± standard deviation (SD). Statistical significance was set at *p* < 0.05 and *p* < 0.01 was considered as highly significant.

## 3. Results

### 3.1. FFP Supplementation Leads to Lower Fasting Blood Glucose Levels and Improves Glucose Tolerance

**Figure 1:**
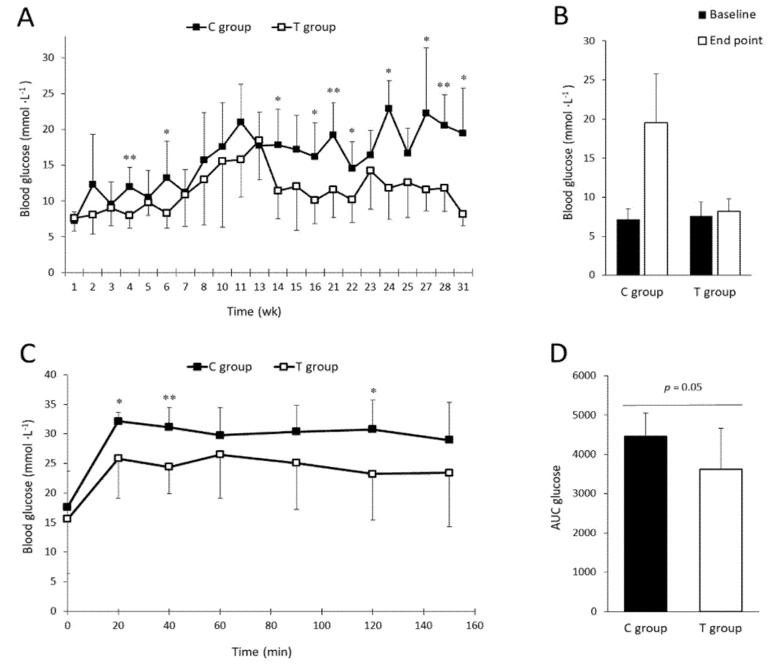
Effect of the fermented food product (FFP) on glucose metabolism. (**A**) Weekly fasting blood glucose (FBG) progression in Zucker diabetic fatty (ZDF) rats. Animals in T group showed lower FBG mean values than animals in C group during the study. Statistically significant differences were found at 4, 6, 14, 16, 21, 22, 24, 27, 28, and 31 weeks (*p* = 0.004, *p* = 0.038, *p* = 0.013, *p* = 0.013, *p* = 0.005, *p* = 0.026, *p* = 0.001, *p* = 0.030, *p* = 0.001, and *p* = 0.016, respectively). (**B**) Bar plots represent basal and end point FBG values in the groups. No statistical significant differences were found between basal and end point FBG values in any groups (7.2 ± 1.3 versus 19.5 ± 6.3 mmol L^−1^ at 0 and 31 weeks, respectively in C group; 7.6 ± 1.8 versus 8.2 ± 1.6 mmol L^−1^ at 0 and 31 weeks respectively in T group). (**C**) Two-months glucose tolerance test (GTT) curve. The GTT curve, after two months of FFP supplementation, showed lower blood glucose levels and statistically significant differences between groups were found at 20, 40, and 120 min (*p* = 0.034, *p* = 0.007, and *p* = 0.038, respectively). (**D**) Area under the curve (AUC) plot. The AUC value was lower in T group (3622.5 ± 1040.4) compared to C group (4454.0 ± 590.9) although the *p* value obtained was in the limit of significance (*p* = 0.05). Values are expressed as mean ± SD. wk = week. * *p* < 0.05, ** *p* < 0.01.

In order to determine the effectiveness of FFP supplementation to control blood glucose levels in ZDF rats supplemented with HD we have performed a FBG determination on ZDF rats fasted during 24 h. No significant differences between groups were observed in FBG values basally (Figure 1A). However, after four weeks of supplementation, animals in the T group exhibited lower FBG values than animals in the C group (8.0 ± 1.8 versus 12.0 ± 2.7 mmol L^−1^, respectively; *p* = 0.004). Statistical significant differences between groups were also found in week six (*p* = 0.038), week 14 (*p* = 0.013), week 16 (*p* = 0.013), week 21 (*p* = 0.005), week 22 (*p* = 0.026), week 24 (*p* = 0.001), week 27 (*p* = 0.03), week 28 (*p* = 0.001), and week 31 (*p* = 0.016) as represented in Figure 1A. Initial and final FBG values were compared in the C group showing no statistical significant differences, despite the observed differences were very high (*p* = 0.125); on the other hand, initial and final FBG values in the T group are comparable and no statistically significant differences were found (*p* = 0.625) (Figure 1B). Although, one-month period was not enough to display significant differences in GTT between groups (data not shown), after two months of supplementation, the T group showed lower glycemic values versus the C group although not all the time points showed significance (Figure 1C). Statistically significant differences were found at GTT time point 20 min (*p* = 0.034), 40 min (*p* = 0.007) and 120 min (*p* = 0.038) time points of the GTT. Glucose AUC showed higher values in the C group with a *p* value in the limit of significance (*p* = 0.05) (Figure 1D). These findings suggest that rats supplemented with the FFP exhibited a better glucose metabolism control than those fed exclusively with HD (C group).

### 3.2. Body Weight, Body Composition, and Food Intake after FFP Consumption

**Figure 2:**
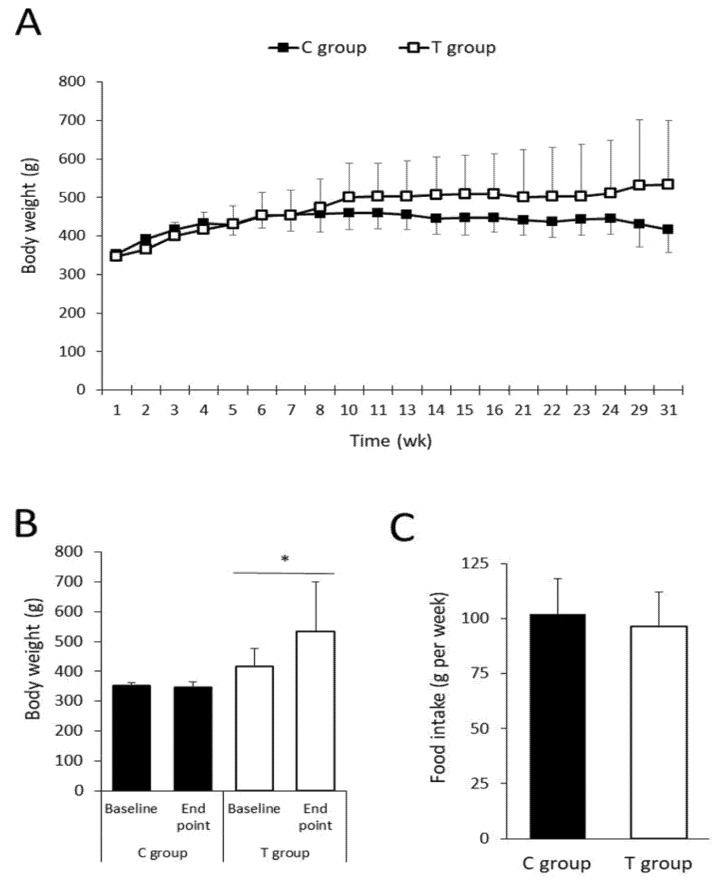
Body weight progression, body weight (BW) gain and average of hypercaloric diet (HD) food intake during the intervention. No statistically significant differences were observed in BW (**A**) between the experimental groups in spite of their divergent growing tendencies. BW gain (**B**) was statistically significant in T group (346.0 ± 18.4 versus 533.5 ± 165.8 g at baseline and at the end point, respectively; *p* = 0.031) but no statistical significant differences were found in C group (352.3 ± 9.9 versus 416.8 ± 60.3 g at baseline and at the end point, respectively; *p* = 0.625). The FFP administration did not alter HD food intake (**C**), which was comparable in both groups (101.9 ± 16.2 versus 96.5 ± 15.7 g in C and T groups, respectively; *p* = 0.413). Values are expressed as mean ± SD. * *p* < 0.05.

Initial body weight values were similar between groups (*p* = 0.610) and BW increase was steady and similar within the groups during the first eight weeks of study, time at which different trends were observed between groups. From week eight onward, the T group continued gaining weight whereas the C group did not increase BW and even a weight loss was observed at the end of the study (Figure 2A).

Regarding the BW gain (difference between end point and basal BW values), statistically significant differences were observed in the T group (*p* = 0.031), while the C group did not experiment statistical significant changes over time (*p* = 0.625) (Figure 2B).

No statistically significant differences were found in HD food consumption between both groups (*p* = 0.413) (Figure 2C). Interestingly, data on food intake differs from the outcomes found in BW, which indicates that animals supplemented with the FFP showed a greater BW gain.

Body composition was evaluated before sacrifice and despite the asymmetry of BW found at the end of the study, no statistically significant differences within groups were assessed in fat mass percentage (*p* = 0.630). Both groups also exhibited similar mean values of lean mass (*p* = 0.641) and other tissues relative percentage (*p* = 0.947) (Figure S3). These results suggest that the supplementation with the FFP did not alter body composition in ZDF rats fed with HD. Supporting the previous presumption, the weights of retroperitoneal, epidydimal, mesenteric, subcutaneous, and brown fat mass collected at sacrifice did not statistically differ between experimental groups (Table S3).

### 3.3. FFP Preserves Normal Metabolic and Biochemical Parameters

Comparisons of lipid profile and hepatotoxicity markers between and within groups are available in Table S4. No statistically significant differences were found at baseline in any of the explored parameters between both experimental groups, except for LDL-C (*p* = 0.021) and TG mean values (*p* = 0.025). During the study both groups experimented an increase in LDL-C values and the magnitude was greater in the C group than in the T group (*p* = 0.001 and *p* = 0.023, respectively). Although baseline TG levels were significantly lower in the C group (*p* = 0.025), a statistically significant increase was observed for the same group after seven months of study (*p* = 0.043) while T group remained unchanged. Indeed, significant differences were appreciated between groups at the end of the trial (*p* = 0.01). Somehow the FFP could restore and normalize TG values. Regarding TC, both C and T groups showed the same tendency of increase at the end of the study (*p* = 0.00 and *p* = 0.011, respectively) and the same response was found in LDL-C (*p* = 0.01 and *p* = 0.023 in the C group and T group, respectively). Taken together, pairwise comparisons between all of the follow-up time points revealed that at the end of the study both groups showed significantly greater serum levels of TC, LDL-C, and HDL.

**Figure 3:**
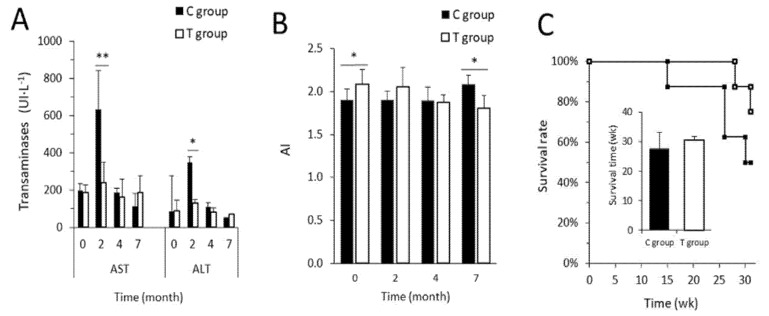
Transaminases level, atherogenic index and survival rate in ZDF rats. (**A**) Transaminase levels. Statistical significant differences were observed in aspartate and alanine amino-transferases (AST and ALT) at two months of study, being that the C group clearly shown higher concentrations of both AST (632.1 ± 208.9 versus 238.9 ± 112.6 UI L^−1^ in C and T groups, respectively; *p* = 0.001) and ALT (348.0 ± 192.2 versus 90.0 ± 54.6 UI L^−1^ in C and T groups, respectively; *p* = 0.019). (**B**) Atherogenic index. Statistically significant differences were observed in basal (1.9 ± 0.1 versus 2.1 ± 0.1 in C and T groups, respectively; *p* = 0.004) and end point values of atherogenic index (AI) (2.1 ± 0.2 versus 1.8 ± 0.1 in C and T groups, respectively; *p* = 0.015). (**C**) Kaplan-Meier plot. The T group demonstrated a greater survival rate (87.5%) compared to the C group (50%). The survival time was higher in the T group (27.6 ± 5.6 versus 30.6 ± 1.1 weeks in C and T groups, respectively, *p* = 0.106) but no significant differences between both groups were found. Data are represented as mean ± SD. * *p* < 0.05; ** *p* < 0.01.

With respect to the liver function and the stress induced by the HD, serum AST and ALT levels were determined along the intervention as well. For both parameters, statistically significant differences were found between T and C groups at two months of study, when a peak on serum AST and ALT was observed: The T group showed significant lower values of both AST and ALT (*p* = 0.001 and *p* = 0.019, respectively). After the aforementioned peak, transaminase levels were normalized, and such reduction was more pronounced in the group supplemented with FFP (Figure 3A). The AI showed statistically significant differences between groups at baseline (*p* = 0.023) and at the end of the study (*p* = 0.020) (Figure 3B). The T group presented a tendency with a better health status in the T group and when pairwise comparison was made, the treated group exhibited an improved and reduced AI at the end of the study in contrast with its baseline value (*p* = 0.027). Regardless the tendency observed in Figure 3B the C group did not present marked differences on AI over time.

Overall, the group supplemented with FFP displayed a healthier phenotype, what is also supported by data on survival rate (Figure 3C). Animals in the T group exhibited a tendency of longer life expectancy (30.6 ± 1.1 weeks) compared to the animals included in the C group (27.6 ± 5.6 weeks) (*p* = 0.106). Throughout the experimental study, five rats died. The deceased animals presented clear T2D symptoms including a maintained hyperglycemia, marked weight loss, greater water consumption (polydipsia), and wetter cage floor due to increased urine excretion (polyuria) [1,38,39] according to the ZDF animal model disease progression [23]. Four of them belonged to the C group (these rats died at week 15, week 26, week 26, and week 30 of study) and only one to the T group (the rat died at week 28 of study). Those results indicate that the supplementation with FFP increase the survival rate at the end of the study (87.5% in the T group) in contrast to the C group (50%).

### 3.4. The Administration of FFP Allows the Maintenance of Normal Pancreatic Activity

**Figure 4:**
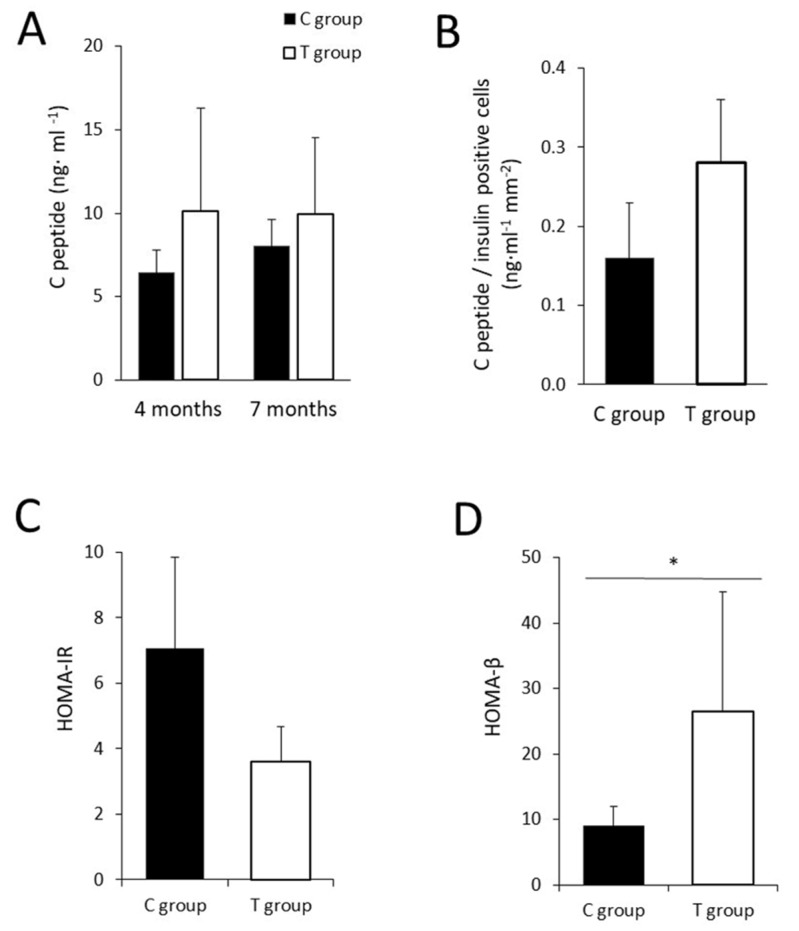
Pancreatic function. (**A**) No statistical significant differences were found between serum C-peptide levels in C and T groups at four months (6.2 ± 1.7 versus 9.1 ± 6.6 ng mL^−1^, respectively; *p* = 0.234) and seven months (7.6 ± 1.7 versus 8.5 ± 4.6 ng mL^−1^, respectively; *p* = 0.792). (**B**) When values of C-peptide/insulin positive cells were determined, a higher value was found in T group although statistical differences were not found (0.2 ± 0.1 versus 0.3 ± 0.1 ng mL^−1^ mm^−2^ in C and T groups, respectively, *p* = 0.114). The homeostatic model assessment (HOMA)-IR (**C**) did not reflect differences among groups, however statistical significant differences were found in HOMA-β (**D**), between T and C groups (26.6 ± 18.2 versus 9.0 ± 2.9, respectively; *p* = 0.029); (*n* = 4 in C group and *n* = 4 in T group). Results are expressed as mean ± SD. * *p* < 0.05.

Blood insulin levels, measured as C-peptide cleavage secretion, were determined at two different time points (four months and seven months) in both groups. The results indicate that although a tendency to protection in the secretion of insulin was observed in the group treated with FFP, no significant differences were found between T group and C group at four and seven months (*p =* 0.234 and *p* = 0.792, respectively) (Figure 4A). Besides, when looking to the ability to synthetize insulin, no significant differences were detected in the total area of insulin-positive cells of pancreatic tissue in both experimental groups (*p* = 0.114). With the aim of evaluating β-cells efficiency in insulin synthesis, we assessed the correspondence between the quantified positive β-cell number and C-peptide levels in serum at the end of the study. The results suggest a higher value of C-peptide levels/β-cell in the T group although no significant differences were found (*p* = 0.114) (Figure 4B).

With respect to the homeostatic model assessment (HOMA) no significant differences were observed in the HOMA-IR (*p* = 0.114). However, statistical significant differences were found in the HOMA-β (*p* = 0.029), remarking a better β-cell functionality in the group supplemented with the FFP (Figure 4C,D).

### 3.5. FFP Supplementation Induces a Decrease in Intestinal Glucose Uptake

**Figure 5:**
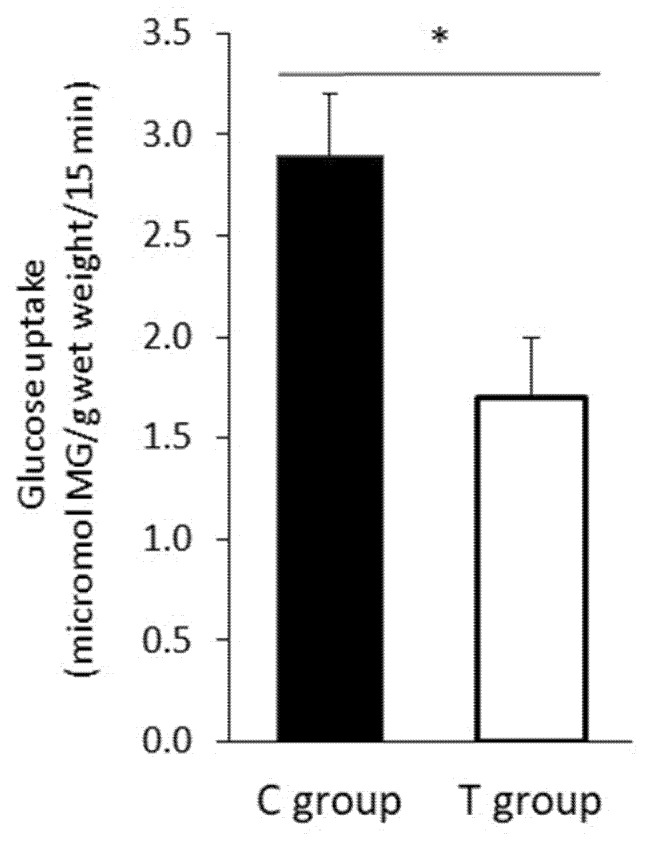
Intestinal glucose uptake. When intestinal glucose transport was determined, it was found that animals administered the FFP (T group) exhibited statistically significant lower marked glucose uptake compared to animals in C group (1.7 ± 0.7 versus 2.9 ± 0.6 micromol MG g wet weight^−1^ 15 min^−1^; *p* = 0.029); (*n* = 4 in C group and *n* = 5 in T group). Results are expressed as mean ± SD. * *p* < 0.05.

To test whether the hypoglycemic actions of FFP supplementation could be related to a decrease in intestinal sugar uptake, we measured the ex vivo α-MG uptake in intestinal everted rings from animals after treatment. A significant decrease in intestinal α-MG uptake was observed in animals receiving FFP compared to the control group (*p* = 0.029) (Figure 5).

### 3.6. The Administration of the FFP Altered the Composition of Faecal Microbiota

**Figure 6:**
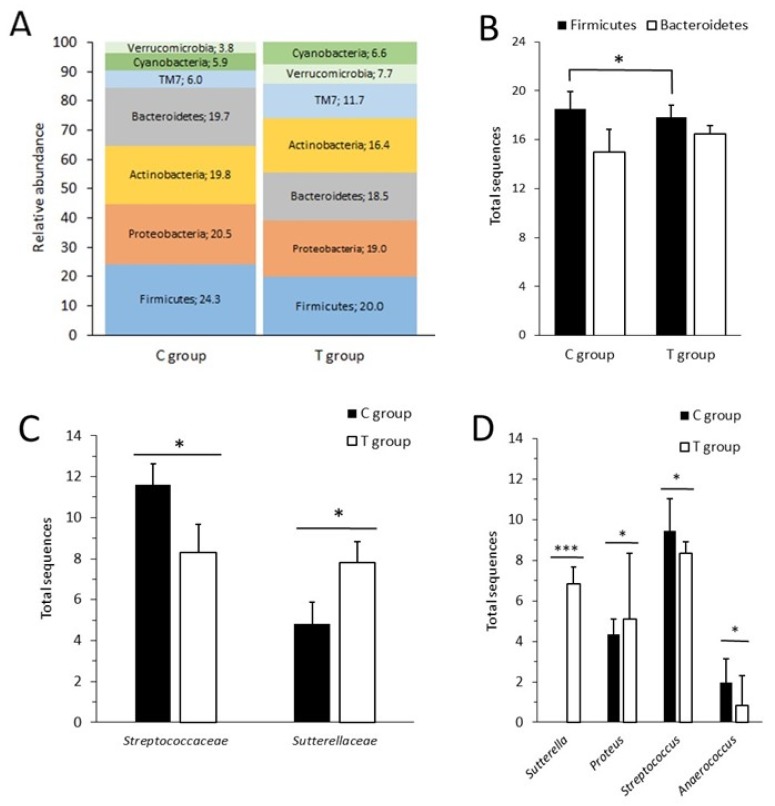
Fecal microbiota composition at phylum, family and genus level. (**A**) Relative abundance of phyla present in fecal samples in C and T groups. (**B**) Contributions of Firmicutes and Bacteroidetes. The abundance of Firmicutes was statistically significant higher in the C group than in the T group (*p* = 0.017) however no statistically significant differences were found in Firmicutes to Bacteroidetes ratio; (*n* = 3 in C group and *n* = 3 in T group). (**C**) Fecal microbiota composition at family level. *Streptococcaceae* contribution was significantly higher in the control rats than in the treated ones while *Sutterellaceae* was enriched in the treated animals; (*n* = 3 in C group and *n* = 3 in T group). (**D**) Widespread effect of the administration of the FFP on bacterial genera. Representation of statistically significant genera between groups at six months. *Sutterella* and *Proteus* were found enriched in the T group whereas *Anaerococcus* and *Streptococcus* were more prominent in the C group; (*n* = 3 in C group and *n* = 3 in T group). * *p* < 0.05, *** *p* < 0.01.

At phylum level, Firmicutes, Proteobacteria, Bacteroidetes, and Actinobacteria were the more predominant phyla present in the fecal samples of both groups (Figure 6A). After FFP supplementation, the percentage of Firmicutes was statistical significantly higher in the C group than in the T group (*p* = 0.017) and no statistical differences were found between groups in the Bacteroidetes phylum (Figure 6B), probably due to the variability observed. The ratio of Firmicutes to Bacteroidetes is a widely used indicator of the microbial composition, however in our study its value was comparable in both groups (1.23 versus 1.08 in C group and T group, respectively). At the family level the more abundant taxa were *Lactobacillaceae* (4.1%), *Enterobacteriaceae* (3.6%), *Porphyromonadaceae* (3.6%), *Lachnospiraceae* (3.5%), and *Ruminococcaceae* (3.4%) (Figure S4). Statistically significant differences were only observed in *Streptococcaceae* (*p* = 0.046) and *Sutterellaceae* (*p* = 0.046) families. The *Streptococcaceae* family was higher in C group while the *Sutterellaceae* was higher in T group (Figure 6C).

The study of the total abundance of the genera revealed that the dominant bacteria genera were *Lactobacillus* (2.85%), *Clostridium* (2.26%), *Bifidobacterium* (2.26%), *Barnesiella* (2.22%), and *Bacteroides* (2.17%). From all 123 different identified genera, four and 19 were found exclusively in C or T groups, respectively (Tables S5 and S6). At genus level, the supplementation with the FFP enriched the abundance of *Sutterella* and *Proteus*, which were found more prominent in the T group (*p* < 0.001 and *p* = 0.032, respectively), while *Anaerococcus* and *Streptococcus* were more copious in control animals (*p* = 0.032 for both genera) (Figure 6D).

**Figure 7:**
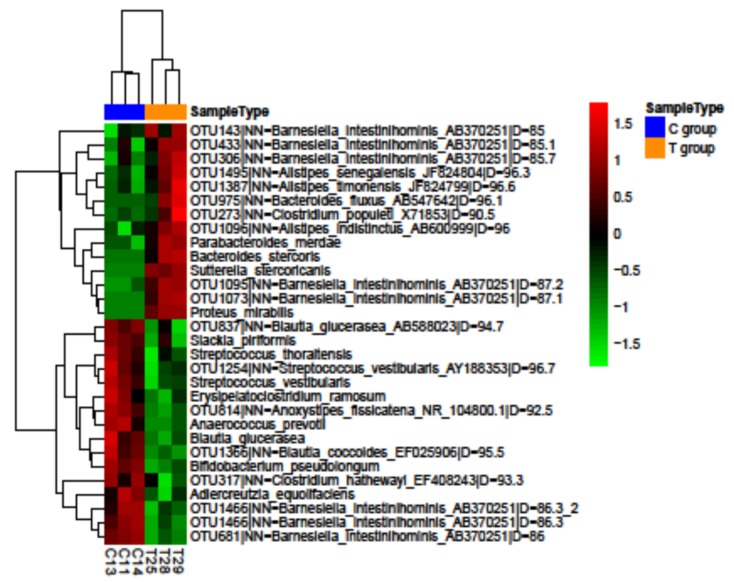
Hierarchical clustering. Hierarchical clustering of differentially abundant species (*p* < 0.05) in C group and T group at six months; (*n* = 3 in C group and *n* = 3 in T group).

A sum of 433 different bacterial species were identified in all the samples, of which 26 and 100 were exclusive of the C and T group, respectively (Tables S5 and S6). A mutual core of 307 shared species was found in the two groups and among them 30 bacterial species significantly differed in the number of total sequences (Figure 7).

Diversity of the fecal microbiota were also analyzed. Alpha diversity indexes of bacterial community in ZDF rats are presented in Table 1. The alpha diversity was greater in T group than in C group, as samples in control group are perceived to have lower value in four of the five analyzed indexes. This may suggest that the administration of the FFP lead to an enrichment of the microbial diversity.

**Figure 8:**
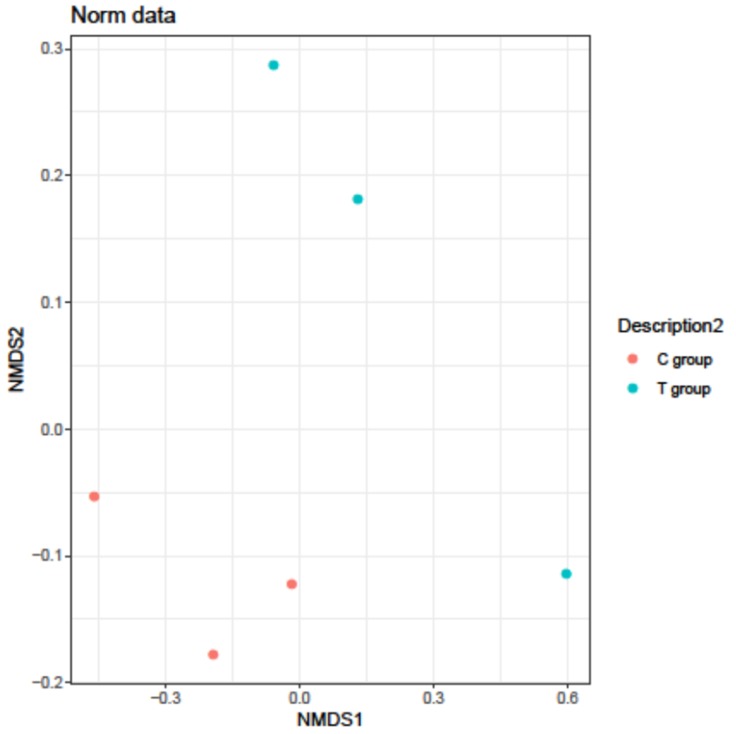
Non-metric multidimensional scaling analysis (NMDS). Representation of samples from C group and T group at six months; (*n* = 3 in C group and *n* = 3 in T group).

A non-metric multidimensional scaling analysis (NMDS) was performed in order to analyze the observed variability. The NMDS plot (Figure 8) showed that the distances between samples from the C group are shorter than those between samples in the T group. This means that C group presents a higher microbial homogeneity and so animals were more similar to each other than the treated animals.

## 4. Discussion

Recent findings regarding T2D have led to new valuable knowledge on diabetic etiology and risk factors. Specifically, considerable interest exists in the role of the GM in the development and progression of diabetes. Several publications showed that diabetic individuals present a characteristic intestinal microbial community. This distinctive GM includes altered abundance of some bacteria taxa, for instance increased presence of *Lactobacillus* genera and opportunistic pathogens, along with differences in metabolic functionality, such as enhanced sulphate reduction and oxidative stress resistance [12]. Moreover, there is evidence that the GM influences the progression of the diabetes and its related complications [40] and its restoration resulted in clinical improvements according to experimental and clinical data. In particular, diet appears to play a key role and nutritional interventions have received attention for their ability to normalize the intestinal microbiota and thus improve health status [41].

In the present study a non-dairy fermented product was tested for its effectiveness in relieving the diabetic phenotype in the ZDF model. Among our experimental data, the markedly decreased glucose absorption in the treated group is probably the most striking result to emerge. Although extreme caution must be taken when extrapolating the results from ex vivo experiments, our result suggests that the total glucose which reach the systemic circulation was strongly attenuated with the FFP supplementation. This would support the lower BFG and GTT values observed in the T group. We hypothesized that the FFP is able to improve the intestinal integrity and to decrease the load of glucose which reach the systemic environment, as suggested by previous authors [42]. A body of evidence has indicated an altered gut environment in diabetic people which includes a compromised tight-junction structure [43], a disrupted gut barrier [40], and impaired glucose transporters in the gut [42]. Taking it all into consideration, any approach that pursue to restore the gut health would eventually induce beneficial changes in the host.

A slightly higher value of C-peptide was also noticed in the supplemented group. The concentration of C-peptide represents an indirect estimation of β-cell activity as it leaks from proinsulin cleavage [44]. On the other hand, although the number of insulin positive cells present in the pancreas of the C group was greater, we observed that β-cells were more efficient in the T group because a higher endogenous insulin production per β-cell was measured. Such findings indicate that the β-cell mass was best preserved in the T group. This is in good agreement with the HOMA-β score, and all together suggests a healthier pancreatic function in the T group. We believe that the treatment indirectly protected from organs dysfunction through a systemic effect, which together with the normalized values of glycemia observed in the T group in both BFG and GTT determinations could be responsible for the better pancreatic activity reported in this group.

Both the restoration of the glucose homeostasis and the protection of the pancreatic activity could be responsible for the improved wellness of the treated ZDF rats. A correct glucose metabolism can lessen alterations in cardio-metabolic parameters and result in a healthier lipid profile. Based on the physiological process of the T2D, also mimicked in the ZDF rat model, a deterioration in the lipid profile would be predicted along with the diabetes progression in both groups. However, our data reflected better values of TG, transaminases, and AI index in the T group. In this context, the observed results may suggest that the FFP helped to some extent to prevent diabetes-associated secondary alterations. Most of the analyzed parameters in the current research showed a tendency of a healthier status in the T group, and this trend was strongly manifested along the study by the greater survival observed in the supplemented group. As a result, the FFP-treated animals showed a prolonged survival in comparison with the control animals. It is also worth noting that the only animal that died in the group treated with FFP did so in week 28, while the first animal that did it in the control group was in week 15.

In addition to the mechanisms outlined above, another feasible explanation for the better glucose homeostasis in the T group is that the GM functional properties were affected by the FFP, and the ability to metabolize glucose was increased in some bacteria groups. Gut dysbiosis has been described in relation to glucose dyshomeostasis and emerging experimental and clinical evidence suggests that GM activity is modified in T2D [12,40,45,46]. Because of the key endocrine function exerted by the GM [47], restoring its metabolic function is a valuable approach which proved to be successful in provoking significative changes [14]. In the metagenomic analysis of the fecal microbiome obtained from the ZDF rats performed in the present study we could identify some differences among groups’ samples and some results did not confirm previous research on the topic. For instance, we did not find significant differences in Firmicutes to Bacteroidetes ratio. It is a relevant but controversial microbial marker. A reduced Bacteroidetes contribution or increased Firmicutes to Bacteroidetes ratio have been reported in obesity [48], and this parameter was found to be partially restored following weight loss [49] or prebiotic treatment [50]. On the contrary, other researchers have reported a significantly higher Firmicutes to Bacteroidetes ratio in lean than in overweighed or obese individuals [51]. In line with this observation, our results indicate a significant higher contribution of Firmicutes in the T group; however, our data did not reveal significant differences in the aforementioned ratio. Unlike other investigations carried out on the topic, we did not find a significant increase in *Bifidobacterium* levels. Based on the characteristics of the FFP, whose composition includes a high level of fermentable carbohydrates [52], a bifidogenic effect was expected in the T group due to the metabolites obtained during the fermentation process of FFP. This change is desired because this bacteria genera is a health marker [53]. However, our results indicate that the analyzed product or the experimental design could not induce a bifidogenic effect.

At the family level, *Streptococcaceae* was found significantly enriched in the control group, which matches with a previous type 1 diabetes (T1D) human trial [54]. On the contrary, *Sutterellaceae* family, which includes commensal species found in healthy human and animals [55], was more predominant in the treated group. At the genus level a significant enrichment was observed in *Sutterella* in the treated group. This finding does not corroborate previous results from earlier case-control studies in prediabetic subjects [56], experimental studies on type 1 diabetic animal models [57] and reports in other pathological conditions such as autism [58] and atopic dermatitis [59]. However, our finding matches to a previous investigation that associated barley consumption, a main component in FFP formulation, with an elevated abundance of *Sutterella* in the human GM [60]. *Proteus* genus was also enriched in the supplemented rats. Although there is no evidence about the function of this genus in diabetes or obesity, some authors suggest its role in some pathological conditions [61,62]. In fact, one specie, *P. mirabilis*, was negatively correlated to health improvements [63,64], what suggests that a low abundance should be wanted. In contrast, our metagenomic analysis revealed that *P. mirabilis* was significantly enriched in the treated group. What is more, it was completely absent in control animal samples. We have no explanation for this striking result. Regarding the C group, *Anaerococcus* and *Streptococcus* genera were found more prominent. A previous report confirmed a lower relative abundance of the former genera in diabetic compared to healthy adults [65], contrary to our results. The latter genera, however, presented a greater relative abundance in prediabetic [56], type 2 diabetic [66], and type 1 diabetic individuals [67] compared to their healthy controls, what matches to our findings.

Among the 30 bacteria species statistically different between groups, eight *Barnesiella spp.* were identified and five of them were greater in the treated group. A previous experimental study reported an increased abundance of this genus in obese mice supplemented with prebiotics which experienced a better glucose tolerance and important metabolic improvements [68]. We also discovered that two *Blautia spp.* (*B. coccoides* and *B. glucerasea*) were more prominent in the control animals. This genus belongs to the family *Lachnospiraceae* and includes butyrate producing bacteria (BPB), a group which has been attributed many beneficial effects [69]. It was reported some positive correlations between *Blautia spp*. and microbial products such as long-chain triglycerides [70] and short chain fatty acids (SCFAs) [71], and parameters like BFG, insulin level, HOMA-IR, and weight loss [56,72]. A decreased abundance of *Blautia coccoides*/*Eubacterium rectale* was found in T1D children and was linked to intestinal disintegrity [73], while a randomized crossover study in healthy adults reported a reduced abundance of *Blautia* genus after prebiotic supplementation [74]. These results appear inconclusive. Our data also showed three *Alistipes spp.* enriched in the group supplemented with the fermented product. On the contrary, previous works reported its enrichment in T1D [67] and T2D individuals [12]. In the same way, our data revealed a lower abundance of two *Streptococcus spp.* (*S. thoraltensis* and *S. vestibularis*) in the group which exhibited a better glucose control (T group) and refute previous research [66].

The relative abundance of *Anaerococcus*, genus which includes many bacteria species which produce butyrate in experimental conditions [75], was significantly enriched in the treated group. Metagenome-wide association studies (MGWAS) on T2D humans revealed a compromised gut health with an abnormal abundance of BPB, thus an enrichment of the aforementioned bacteria group could, hypothetically, have led to improvements in the health status of the T group [12,46]. Regrettably, this study did not confirm previous research and the treatment with the FFP did not induce changes in well described BPB *spp* such as *Akkermasia muciniphila. A. muciniphila* is a mucus producer belonging to the *Verrucomicrobiae* family which has been hypothesized to protect against T2D and obesity in animal studies [69,76]. Increased levels of *A. muciniphila* were correlated to improvements in health parameters in healthy [77,78] and HD-fed mice [76], and previous works in healthy mice showed that dietary manipulation such as supplementation with prebiotics could increase the abundance of intestinal *A. muciniphila* [79]. It seems that the nutritional properties of the FFP and/or the study design did not allow for a greater presence of *A. muciniphila* in the GM.

Regardless microbial populations, microbial metabolites are also of great importance and could be partly responsible for the improved glucose control and gut health found in the T group. Considerable interest exists in SCFAs such as acetate, propionate, and butyrate. They are lipid molecules known to mediate in inflammation [80], gut permeability [81], energy expenditure and metabolism [51,82], and an insulin-sensitizing [83] and antidiabetic effects [84,85] have also been described for them. Taking it into consideration, the promotion of their physiological levels can lead to an improved overall health, and may partly explain the favorable phenotype reported in the treated animals.

Since the FFP is rich in factors associated to a reduced food ingestion, such as fiber, microbial metabolites, and probiotic LABs [86,87,88], a decreased food intake was expected in the treated group. However, no considerable differences in eating behavior were observed between groups. This unexpected result is in good agreement with other previous studies with fermented dairy product in STZ-induced diabetic rats [25]. Similarly, BW was not significantly changed during the supplementation and was indeed more prominent on the T group. Even though these results differ from an earlier in vivo study [89] and a crossover trial in prediabetic humans [18] that found a protection from weight gain for fermented products, they are consistent with previous finding in ZDF rats and other animal models of T2D [90,91]. Although no anti-obesity effect was observed for the FFP, it remains unknown whether it has some effect on body gain. May studies on another obesity rodent’s model elucidate the impact of FFP on body mass regulation such as the ob/ob mouse model. The unexpected dramatic reduction in BW in the C group could be consequence of an acute failure of the pancreatic function. The decline in C-peptide levels in control rats suggest a low serum insulin level which, along with a compromised insulin signaling would promote lipolysis and favor fat mobilization from the tissue [92,93]. In much the same way, a FFP-mediated increase of SCFA levels in the T group, microbial metabolites known to downregulate lipolysis [94,95,96], could have prevented from the loss of weight in the treated ZDF rats. A previous report on Monascus fermented rice concluded that the differences in weight loss between control versus treated animals could be caused by a depletion in the lean mass in the former as results of diabetic complications [90]. In the present research, however, the nuclear magnetic resonance revealed the absence of discrepancies in body composition after FFP supplementation. It differs from previous results on fermented food in controlled intervention studies [18,97] and animals models [98] which evidenced a significant decrease in body weight and fat depots. Nevertheless, the absence of significant changes in body composition did not abstain from improvements in glucose homeostasis, what matches well with previous findings in murine models of obesity [99,100].

With reference to the microbial diversity, the results of the analysis strongly indicate that the FFP supplementation led to a greater diversity in the microbial communities which inhabit the gut environment of the ZDF rats. Another plausible explanation may be that the FFP abstained from the loss of diversity which consorts some clinical disorders such as obesity [48], however some controversy does exist with regards to T2D [12]. It is well-known that dietary factors, along with other external agents, have a great importance for the diversity and composition of the GM [101,102,103]. Previous works already studied changes in alpha-diversity with the consumption of diverse functional compounds. For instance, a meta-analysis compared different prebiotic treatments, and their efficacy in increasing bacteria richness was found to be dependent on the fiber used and the diversity index calculated. The authors concluded that fiber interventions did not increase alpha-diversity [104]. They however suspected that a longer exposition would reveal some differences, what is in good agreement with our findings. Further, other authors reached the conclusion that the bigger the diversity of the GM, the bigger its resilience against external challenges [13]. This might consequently provide a healthier phenotype since a low bacteria richness was found to be present in illnesses and pathological conditions [103,105] and may this stability explain the better status reported for the treated group in our study. However, a compromised gut richness is not always present on T2D according to findings from a MGWAS with diabetic individuals [12] and bacterial diversity should not be the only focal point. Some authors call for a deeper approach and suggest that GM metabolic functionality, and not only its phylogenetic composition, could be an interesting target for future research and shed light on this point [106]. Then a more comprehensive approach is recommended.

This is not the first study reporting a hypoglycemic effect of probiotic bacteria or fermented products in animal models, however, there is a critical need for well-designed, controlled studies in humans to provide solid evidence of the suitability of fermented food for T2D management or prevention. Although the evidence from controlled trials in humans is limited and arises from small sample sizes [18,107], the number of work assessing the antidiabetic effects of fermented products in humans continues to grow [98,108]. Different fermented food products were investigated for their ability to exert antidiabetic effects and a wide variety of outcomes and levels of scientific evidence were reported [27]. On account of the fact that the study sample was small, and it was a preliminary attempt to test the FFP on an in vivo model, we strongly believe that a bigger sample size would have evidence more differences in the analyzed parameters. Nevertheless, previous supplementation with live bacteria in a different T2D murine model also failed to find significant differences in some metabolic markers and it did not abstain from relevant beneficial effects in pancreatic function and glucose homeostasis [109]. Nonetheless, the present research presents a valuable characteristic regarding its experimental design. In contrast to some reports in the literature in which the supplementation lasted for a few weeks [18,90,97], we supplemented the rats for a longer time period (31 weeks). This prolonged exposition allowed us the examination of long-term responses.

Notwithstanding, as discussed above, we strongly believe that more work is needed to further understand how FFP works and thereafter, validate its potential effectiveness in diabetic patients. A future double-blind, placebo-controlled study with T2D individuals is being considered and would provide insight into the potential antidiabetic properties of FFP in humans. As not all the strains belonging to the same specie shares exactly the same beneficial effect [28,29], probably because of tiny differences in their physical and chemical properties [110], a full and comprehensive identification of the multi-species consortia of microorganisms presents in FFP, preferably up to strain level [111], would be of great important for it further characterization.

## 5. Conclusions

In summary, we demonstrated that the FFP was favorable on glucose metabolism and contributed to health maintenance, abstained from T2D harmful effects and improved overall life expectancy. Our study is in line with previous studies showing that modulation of GM can confer health benefits on T2D and OB. However, it is a fundamental issue to determine which component(s) present in the FFP is/are responsible for the observed beneficial effects. Research into solving this dilemma is already underway and we hope we could elucidate this issue. Future works on the topic would clearly be worth pursuing. Importantly, the undeniable disparities between experimental models and humans challenge the extrapolation of data from in vivo studies to humans, and a large well-controlled trial with an appropriate study design and statistical methods is needed to provide firm evidence of FFP’s antidiabetic properties.

These findings spotlight once again the role of microorganisms and gut function on the diabetic pathology and indicate that novel fermented products could be a powerful tool to protect against metabolic alterations. Nevertheless, very few publications are available in the literature that address the application of fermented products in diabetic humans and discordance within conclusions makes difficult the elucidation of reliable markers. Our results open the possibility to explore the effectivity of innovative fermented food products in T2D, OB, and other non-communicable diseases in a near future.

## Figures and Tables

**Table 1:** Ecological indexes of Alpha diversity.

Sample	ACE	Chao1	Observed Species	Shannon	Simpson
C group	2235.43 ± 314.85	2280.57 ± 322.74	1900.00 ± 298.08	4.99 ± 0.72	0.98 ± 0.01
T group	2837.85 ± 668.01	2868.51 ± 646.40	2510.67 ± 655.89	5.04 ± 0.76	0.98 ± 0.02

Values expressed as mean ± SD.

## Supplementary Materials

The following are available online at https://www.mdpi.com/2072-6643/11/10/2530/s1, Figure S1: Image of the FFP, Figure S2: Experimental design, Figure S3: Body composition at the time of sacrifice measured by nuclear magnetic resonance (NMR), Figure S4: Relative abundance of fecal microbiota at the family level, Table S1: Culturable and viable counts determined in the FFP, Table S2: Composition of FFP and ENVIGO TD.06416 hypercaloric diet, Table S3: Body fat weight for rats in C and T groups, Table S4: Follow-up of lipid profile in ZDF rats, Table S5: List of bacteria species and OTUs which were identified exclusively in the C group, Table S6: List of bacteria species and OTUs which were identified exclusively in the T group.
