# Supplementary material for: A Fermented Food Product Containing Lactic Acid Bacteria Protects ZDF Rats from the Development of Type 2 Diabetes
Source: Nutrients. 2019 Oct 20;11(10):2530. doi: 10.3390/nu11102530 (PMC6835361; doi:10.3390/nu11102530)
Supplement: Supplementary file 1 [file nutrients-11-02530-s001.pdf]

Electronic supplementary Information

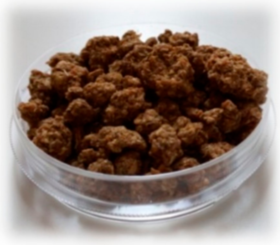

Figure S1. Image of the FFP. The FFP is presented as a dry granulated product, with an average particle size ranging from 4 to 12 mm with a moisture content of 12.8% and a pH of 4.4. FFP: fermented food product.

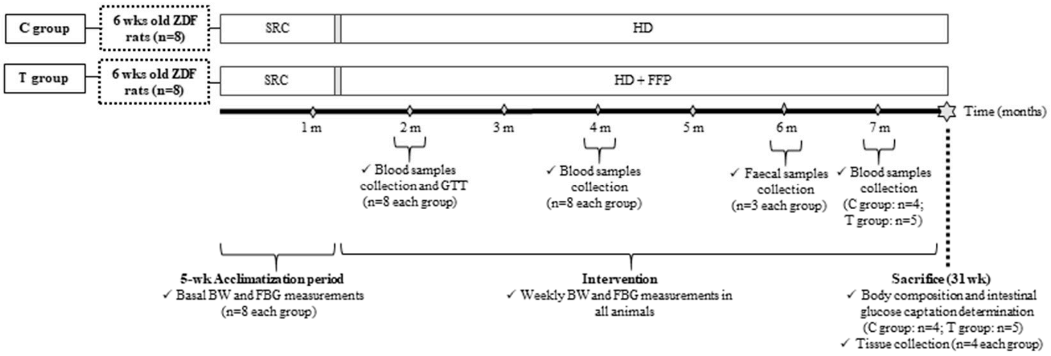

Figure S2. Experimental design. BW: body weight; FBG: fasting blood glucose; FFP: fermented food product; GTT: glucose tolerance test; HD: hypercaloric diet; SRC: standard rodent chow.

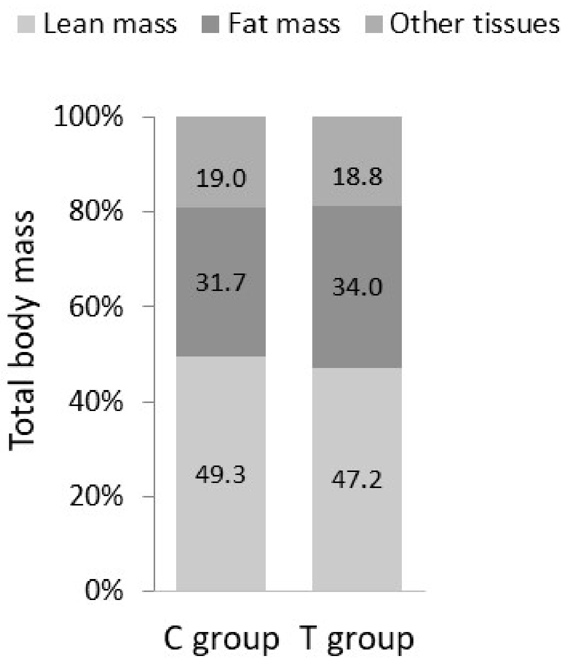

Figure S3. Body composition at the time of sacrifice measured by nuclear magnetic resonance (NMR). The results are expressed as relative contribution of fat mass, lean mass and other tissues. No statistical differences were observed between C and T group in lean mass (49.3 ± 3.9 *vs*. 47.2 ± 7.7 % respectively; p=0.641), fat mass (31.7 ± 3.5 *vs*. 34.0 ± 8.4 % respectively; p=0.630) and other tissues (19.0 ± 5.6 *vs*.18.8 ± 3.5 % respectively; p=0.947).

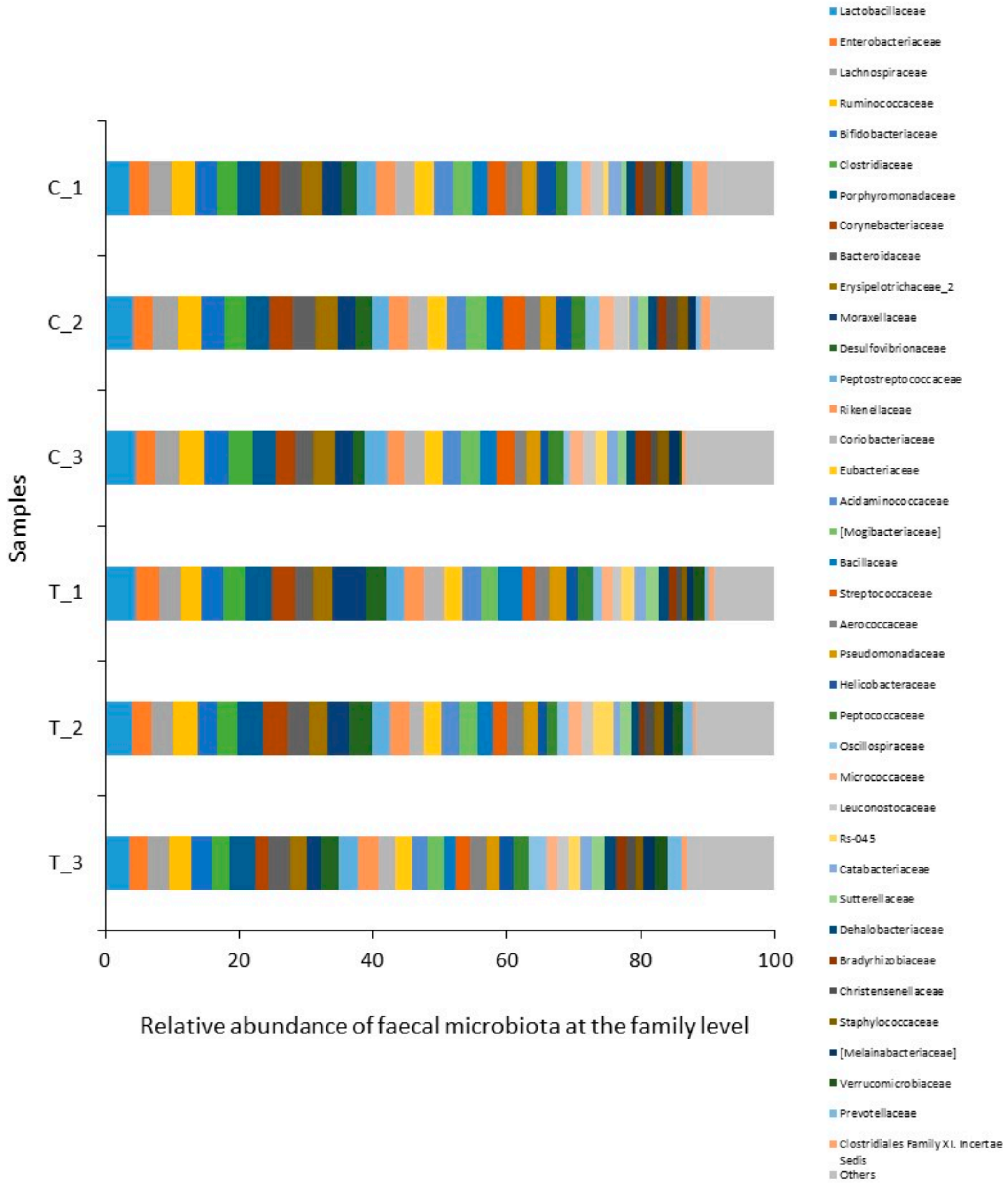

Figure S4. Relative abundance of faecal microbiota at the family level. C_1, C_2 and C_3 are samples of C group and T_1, T_2 and T_3 are samples of T group.

Table S1. Culturable and viable counts determined in the FFP. CFU: colony-forming unit; FFP: fermented food product.

Media and conditions	Target microorganisms	Viable counts (CFU/g)
Trypticase soy agar, 37ºC for 24-48h, aerobiosis	Total bacteria	2.0 x 10^5
Man, Rogosa and Sharpe agar, 37ºC for 24-48h, anaerobiosis	Lactobacilli	4.6 x 10^7
Potato dextrose agar, 25ºC for 2-4 days, aerobiosis	Yeast and fungi	1.0 x 10^5

Table S2. Composition of FFP and ENVIGO TD.06416 hypercaloric diet (per 100 g of product). FFP: fermented food product.

Components	FFP	TD.06416
Calories (kcal)	467.6	510.0
Fats (%)	2.4	10.2
Proteins (%) (Nx6.25)	44.5	20.0
Carbohydrates (%)	53.1	69.8

Table S3. Body fat weight for rats in C and T groups. No statistical significant differences between groups were found in the weight of the different fats. The results are expressed as the mean value ± SD. g: grams.

	C group	T group
Body fat (g)	147.3 ± 42.0	163.6 ± 77.5
Body fat BW^{-1} (%)	31.7 ± 3.5	34.0 ± 8.4
Retroperitoneal fat (g)	16.3 ± 6.5	28.5 ± 16.1
Epidydimal (g)	9.5 ± 1.7	13.5 ± 4.0
Mesenteric fat (g)	4.6 ± 0.7	5.1 ± 2.1
Subcutaneous fat (g)	15.5 ± 5.3	24.7 ± 12.1
Brown fat (g)	0.4 ± 0.2	0.9 ± 0.4

Table S4. Follow-up of lipid profile in ZDF rats. Means in the same column with different letters A, B or C and the same row with different letters a or b differ significantly (*p*< 0.05). n=8 animals/group. Data are expressed as mean ± SD. ZDF: Zucker diabetic fatty; TC: serum total cholesterol; HDL-C: high-density lipoprotein cholesterol; LDL-C: low-density lipoprotein cholesterol; TG: triglycerides.

	Time (month)	C group	T group
TC (mg dL^{-1})	0	155.3 ± 11.7^{Aa}	143.3 ± 10.8^{Aa}
	2	395.1 ± 55.6^{BCa}	353.9 ± 95.2^{Ba}
	4	380.5 ± 35.3^{Ba}	342.1 ± 77.6^{Ba}
	7	478.4 ± 61.2^{Ca}	424.7 ± 147.0^{Ba}
LDL-C (mmol L^{-1})	0	0.5 ± 0.1^{Aa}	0.3 ± 0.1^{Ab}
	2	2.2 ± 0.6^{Ba}	1.5 ± 1.2^{Ba}
	4	2.4 ± 0.5^{Ba}	2.1 ± 0.9^{Ba}
	7	3.4 ± 0.8^{Ca}	2.9 ± 2.0^{Ba}
HDL-C (mmol L^{-1})	0	2.8 ± 0.5^{Aa}	2.5 ± 0.4^{Aa}
	2	6.5 ± 0.7^{Ba}	5.4 ± 2.0^{Ba}
	4	6.5 ± 0.8^{Ba}	6.5 ± 1.3^{Ba}
	7	6.3 ± 2.1^{Ba}	7.2 ± 2. 1^{Ba}
TG (mg dL^{-1})	0	223.4 ± 44.8^{Aa}	311.5 ± 95.7^{Ab}
	2	530.1 ± 129.0^{Ba}	585.9 ± 135.3^{Ba}
	4	531.3 ± 162.4^{Ba}	481.9 ± 90.2^{Ba}
	7	791.0 ± 267.4^{Ba}	452.7 ± 148^{ABb}

Table S5. List of bacteria species and OTUs which were identified exclusively in the C group.

OTU1562|NN=Clostridium_glycolicum_AY007244|D=96.5

Clostridium_celatum
OTU1634|NN=Blautia_stercoris_HM626177|D=94.7
OTU407|NN=Soleaferrea_massiliensis_JX101688|D=87.4
Clostridium_methylpentosum
OTU270|NN=Dorea_massiliensis_JX101687|D=94.1
OTU1336|NN=Clostridium_methylpentosum_Y18181|D=91.5_2
OTU997|NN=Catabacter_hongkongensis_AB671763|D=82.9
OTU566|NN=Clostridium_hathewayi_EF408243|D=97
Abiotrophia_para-adiacens
Citrobacter_werkmanii
Staphylococcus_schleiferi
OTU1353|NN=Clostridium_citroniae_DQ279737|D=92.5
OTU1178|NN=Papillibacter_cinnamivorans_AF167711|D=89.4
OTU307|NN=Sphingobacterium_multivorum_KF535155.1|D=91.3
OTU1282|NN=Roseburia_faecis_AY804149|D=92.8
Abiotrophia_defectiva
OTU1739|NN=Enterobacter_cancerogenus_JN644583|D=96.9
OTU1347|NN=Eubacterium_dolichum_AB649277|D=77.1
OTU1201|NN=Blautia_glucerasea_AB588023|D=94.1
Propionibacterium_acidipropionici
OTU155|NN=Sphingomonas_panni_AJ575818|D=96.7
OTU1006|NN=Eubacterium_rectale_AY804151|D=93.3
Candidatus Stoquefichus_massiliensis
Blastococcus_massiliensis
OTU1222|NN=Clostridium_sporosphaeroides_CLORR16SAD|D=91.2

Table S6. List of bacteria species and OTUs which were identified exclusively in the T group.

OTU1073|NN=Barnesiella_intestinihominis_AB370251|D=87.1
Bacteroides_stercoris
Sutterella_stercoricanis
Lysinibacillus_massiliensis
Proteus_mirabilis
OTU975|NN=Bacteroides_fluxus_AB547642|D=96.1
OTU1478|NN=Ruminococcus_flavefaciens_AY349157|D=94
OTU69|NN=Oscillibacter_valericigenes_AB238598|D=94.8
OTU883|NN=Clostridium_lactatifermentans_AY033434|D=90.6
Tyzzerella_lactatifermentans
OTU193|NN=Barnesiella_intestinihominis_AB370251|D=85.1
OTU60|NN=Ruminococcus_bromii_DQ882649|D=89.6
OTU217|NN=Gemmiger_formicilis_GU562446|D=96.5
OTU521|NN=Papillibacter_cinnamivorans_AF167711|D=89.6
OTU1242|NN=Eubacterium_cellulosolvens_AY178842|D=93.3
OTU815|NN=Pseudomonas_monteilii_GQ284481|D=96.2
OTU478|NN=Butyricicoccus_pullicaecorum_EU410376|D=91.9
OTU267|NN=Parasutterella_excrementihominis_AB370250|D=92.5
OTU779|NN=Lysinibacillus_sphaericus_AJ311894|D=94.4
OTU1337|NN=Desulfovibrio_desulfuricans_DVURRDA|D=89
OTU1101|NN=Clostridium_lactatifermentans_AY033434|D=94
OTU1735|NN=Pseudomonas_monteilii_GQ284481|D=96.6
OTU1212|NN=Ruminococcus_lactaris_NR_027579.1|D=87.5
OTU1381|NN=Pseudomonas_fluorescens_KJ161327|D=96.2
OTU655|NN=Clostridium_asparagiforme_AJ582080|D=89.7_2
OTU45|NN=Soleaferrea_massiliensis_JX101688|D=88.7
OTU814|NN=Anoxystipes_fissicatena_NR_104800.1|D=92.5_2
Odoribacter_laneus
Lachnoclostridium_indolis
OTU1508|NN=Eubacterium_rectale_AY804151|D=94.1
OTU328|NN=Eubacterium_ventriosum_EUBRRDAB|D=94.6
Bifidobacterium_longum
OTU522|NN=Melainabacter_A1|D=92.9
OTU1330|NN=Clostridium_lactatifermentans_AY033434|D=90.5
OTU1119|NN=Soleaferrea_massiliensis_JX101688|D=89.3
Actinomyces_canis
OTU1475|NN=Clostridium_clariflavum_NR_102987.1|D=83
OTU814|NN=Anoxystipes_fissicatena_NR_104800.1|D=92.5_3
OTU1570|NN=Clostridium_clariflavum_NR_102987.1|D=79.3
OTU1722|NN=Clostridium_clariflavum_NR_102987.1|D=83.6
OTU239|NN=Adlercreutzia_equolifaciens_AB306660|D=95
OTU444|NN=Ruminococcus_albus_AY445596|D=91.1
OTU315|NN=Clostridium_clostridioforme_AY169422|D=91.7
OTU722|NN=Oscillibacter_valericigenes_AB238598|D=91.6
OTU944|NN=Intestinimonas_butyriciproducens_JX101685.1|D=91.5
OTU1424|NN=Dorea_formicigenerans_EUBRRDP|D=96.7
OTU1108|NN=Clostridium_bolteae_AJ508452|D=92.8
OTU1598|NN=Oscillospira[Pseudoflavonifractor]_capillosus_AY136666|D=90.1
OTU355|NN=Clostridium_lactatifermentans_AY033434|D=90.6
Psychrobacter_arenosus
OTU1115|NN=Coprococcus_comes_EF031542|D=95
OTU42|NN=Ruminococcus_gauvreauii_EF529620|D=88.4
OTU891|NN=Blautia_coccoides_EF025906|D=93.1
Desulfovibrio_fairfieldensis
OTU523|NN=Barnesiella_intestinihominis_AB370251|D=82_2
Neisseria_macacae
OTU744|NN=Ruminococcus_callidus_X85100|D=91.1
OTU1427|NN=Clostridium_clariflavum_NR_102987.1|D=82.3
OTU523|NN=Barnesiella_intestinihominis_AB370251|D=82
OTU1597|NN=Eubacterium_sulci_AJ006963|D=89.4
Alistipes_finegoldii
OTU1461|NN=Desulfitobacterium_frappieri_DFU40078|D=84.5
Neisseria_elongata
Lactococcus_garvieae
OTU1319|NN=Roseburia_intestinalis_AB661435|D=88.7_2
OTU1391|NN=Clostridium_clariflavum_NR_102987.1|D=84
OTU1704|NN=Clostridium_sporosphaeroides_CLORR16SAD|D=91.6
OTU254|NN=Stenotrophomonas_rhizophila_AB539813|D=96.6
OTU661|NN=Blautia_wexlerae_EF036467|D=94.9
Pediococcus_damnosus
Pseudomonas_oleovorans
OTU1116|NN=Clostridium_hathewayi_EF408243|D=93.2
Blautia_torques
OTU167|NN=Soleaferrea_massiliensis_JX101688|D=88.5
OTU570|NN=Ruminococcus_gauvreauii_EF529620|D=91.2_4
OTU82|NN=Clostridium_lactatifermentans_AY033434|D=93.1
OTU930|NN=Brevibacillus_agri_AY319301.1|D=78.3
Brevundimonas_vesicularis
Bacillus_megaterium
Microbacterium_phyllosphaerae
Lactococcus_lactis
OTU184|NN=Ochrobactrum_anthropi_KC845230|D=79
OTU446|NN=Brachybacterium_paraconglomeratum_EU660345|D=96.1
Stenotrophomonas_maltophilia
OTU1064|NN=Leuconostoc_gelidum_KF577567|D=96.8
Lactobacillus_paracasei
Butyrivibrio_crossotus
Catabacter_hongkongensis
OTU1487|NN=Melainabacter_A1|D=96.1
OTU152|NN=Oscillibacter_valericigenes_AB238598|D=94.3
OTU1546|NN=Soleaferrea_massiliensis_JX101688|D=87.8
OTU175|NN=Roseburia_hominis_AB661434|D=94.3
OTU287|NN=Coprococcus_catus_AB361624|D=96.2
OTU429|NN=Clostridium_methylpentosum_Y18181|D=81.9_2
OTU644|NN=Dorea_massiliensis_JX101687|D=88.1
OTU821|NN=Massilia_aurea_AM231588|D=96.5
OTU823|NN=Clostridium_bartlettii_AY438672|D=96.4
OTU826|NN=Lactobacillus_vaginalis_GQ422709|D=94
Serratia_marcescens
Streptococcus_parauberis
Tyzzerella_lactatifermentans
